# De novo mutations in autosomal recessive congenital malformations

**DOI:** 10.1038/gim.2016.62

**Published:** 2016-06-09

**Authors:** Holly A. Black, David Parry, Santosh S. Atanur, David Ross, Elaine Rose, Helen Russell, Sarah Stock, Jon Warner, Mary Porteous, Timothy J. Aitman, Margaret J. Evans

**Affiliations:** 1 Centre for Genomic and Experimental Medicine, Institute of Genetics and Molecular Medicine, University of Edinburgh, Edinburgh UK; 2 NHS Fife Fetal Medicine Unit, Victoria Hospital, Kirkcaldy, UK; 3 Tommy’s Centre for Maternal and Fetal Health, MRC University of Edinburgh Centre for Reproductive Health, The Queen’s Medical Research Institute, Edinburgh UK; 4 South East of Scotland Clinical Genetics Service, Western General Hospital, Edinburgh UK; 5 Department of Pathology, NHS Lothian, Edinburgh UK

**Keywords:** Mutation, Disease genetics, Diagnosis, Prognosis

**To the Editor:** In suspected autosomal recessive disease without a genetic diagnosis, the risk to future pregnancies is generally reported as 25%, assuming inheritance of one causative allele from each parent. However, it is also possible that a de novo mutation (DNM) could contribute to disease in these cases. The contribution of DNMs to autosomal dominant developmental disorders is widely recognized, but their frequency in autosomal recessive disease is thought to be low, as found in a recent *Genetics in Medicine* article by Retterer et al.,^1^ titled “Clinical Application of Whole-Exome Sequencing Across Clinical Indications,” and other studies.^2,3^

The genetic diagnosis of such conditions is important because it allows precise understanding of disease etiology and accurate reporting of recurrence risk. This enables families to make informed decisions about future reproduction and provides the opportunity for earlier genetic screening in future pregnancies. Without a genetic diagnosis, recurrence risk is estimated on the basis of the clinical phenotype alone.

In a series of nine families in which severe fetal malformation led to the termination of a pregnancy, we used trio-based exome sequencing to identify two unrelated cases in which an autosomal recessive disorder was caused by the combination of one de novo and one inherited variant. Compound heterozygous loss-of-function variants in *EVC2* were identified in the first fetus. Variants in this gene have been shown to cause Ellis-van Creveld syndrome, which was consistent with the phenotype of the fetus. The first variant was a 1-bp deletion in exon 16 (ENST00000344408.5:c.2746delA) that results in a frameshift generating a premature stop codon (ENSP00000342144.5: p.Ser916AlafsTer6). This was inherited from the mother. The second was a de novo nonsense variant in exon 18 (ENST00000344408.5:c.3141G>A; ENSP00000342144.5:p.Trp1047Ter), which allele-specific polymerase chain reaction confirmed had arisen on the paternal haplotype.

In the second family, compound heterozygous loss-of-function variants in *FRAS1* were identified in the fetus; these mutations cause Fraser syndrome, which was consistent with the phenotype. The first variant was a 20-bp deletion in exon 41 (ENST00000264895.6:c.5664_5665+19delinsT), leading to the loss of two bases of coding sequence and a donor splice site, which is predicted to lead to skipping of exon 41 and a frameshift in the open reading frame, generating a premature stop codon (ENST00000264895.6:p.Asp1845ThrfsTer10). This variant was inherited from the mother. The second variant was a de novo 1-bp deletion in exon 66 (ENST00000264895.6:c.10287delC), resulting in a frameshift mutation and a premature stop codon (ENSP00000264895.6:p.Tyr3429Ter). Allele-specific polymerase chain reaction confirmed that the DNM had arisen on the paternal chromosome.

The variants were validated by Sanger sequencing, and the biological relationships within both trios were confirmed, showing that these were true DNMs. For these two fetuses, the clinical phenotypes were suggestive of autosomal recessive syndromes with a reported recurrence risk of 25%. In both cases, because one of the causative variants arose de novo, the recurrence risk is markedly reduced, with significant implications for future reproductive decisions. A small finite risk remains, however, owing to the possibility of germ-line mosaicism in the father in both families.

Three recently published trio-based exome sequencing studies, each including several thousand cases, a proportion of which presented as congenital malformations, did not report a single case of recessive disorder in which one of the causative alleles was a DNM.^1,2,3^ Retterer et al.^1^ reported exome sequencing in more than 3,000 cases and Yang et al.^2^ in more than 2,000 cases; together these studies report more than 400 autosomal recessive molecular diagnoses, without a single case of a DNM contributing to the genetic etiology. The Deciphering Developmental Disorders project did not identify enrichment for any functional class of DNMs in autosomal recessive developmental disorder–linked genes.^3^ Although our study size is small, it suggests that DNMs may have a more important role in autosomal recessive congenital malformation than has previously been considered.

Given that the identification of a causative DNM in recessive disease leads to a low predicted recurrence risk but has hitherto been infrequently reported, we recommend that the frequency of this mode of inheritance be analyzed systematically in a larger series of cases, particularly in patients presenting with a congenital malformation. Future studies of recessive disease should specifically report whether this mechanism contributes to any of their cases. For accurate clinical reporting, this mode of inheritance should always be considered a possible contributor to the cause of autosomal recessive congenital malformation. Clinical confirmation of parental carrier status will help to ensure accurate reporting of recurrence risk in autosomal recessive disease.

## Disclosure

T.J.A. is in receipt of speaker honoraria from Illumina and consultancy fees from AstraZeneca. The other authors declare no conflict of interest.
